# Endometrial preparation and maternal and obstetrical outcomes after frozen blastocyst transfer

**DOI:** 10.1016/j.xagr.2022.100081

**Published:** 2022-08-07

**Authors:** Kazumi Takeshima, Kenji Ezoe, Sachie Onogi, Nami Kawasaki, Hiroko Hayashi, Tomoko Kuroda, Keiichi Kato

**Affiliations:** Kato Ladies Clinic, Tokyo, Japan.

**Keywords:** clomiphene citrate, congenital anomalies, frozen blastocyst transfer, hormone replacement cycle, natural cycle, pregnancy complications

## Abstract

**BACKGROUND:**

Two types of endometrial preparation protocols are used for frozen embryo transfers in current practice: hormone replacement and the natural cycle. Endometrial preparation in the natural cycle reportedly increases the chances of live birth and decreases early pregnancy loss compared with that in the hormone replacement cycle. However, the influence of endometrial preparation on maternal and neonatal health remains unclear.

**OBJECTIVE:**

This study aimed to investigate whether the differences between hormone replacement cycle and natural cycle influence perinatal outcomes and risk of congenital anomalies in frozen-thawed blastocyst transfer fetuses or births.

**STUDY DESIGN:**

Perinatal outcomes and congenital abnormalities were compared between the natural and hormone replacement cycles. According to the timing of ovulation, frozen-thawed blastocyst transfers in the natural cycle were classified into 2 patterns: on day 4.5 (ovulation 4.5) or day 5 (ovulation 5.0) after ovulation. When the serum luteinizing hormone level was not increased on the day of the trigger, a single vitrified-warmed blastocyst transfer was performed on day 7 after the trigger (ovulation 5.0). When the luteinizing hormone level was slightly increased on the day of trigger, single vitrified-warmed blastocyst transfer was performed on day 6 after the trigger (ovulation 5.0). In total, 67,018 cycles (ovulation 4.5, 29,705 cycles; ovulation 5.0, 31,995 cycles; hormone replacement, 5318 cycles) of frozen-thawed blastocyst transfer between January 2008 and December 2017 at Kato Ladies Clinic were retrospectively analyzed. During the study period, embryo cryopreservation was performed using a vitrification method in all cycles.

**RESULTS:**

Hormone replacement cycles were associated with a higher occurrence of hypertensive disorders of pregnancy (adjusted odds ratio, 2.16; 95% confidence interval, 1.66–2.81) and placenta accreta (adjusted odds ratio, 4.14; 95% confidence interval, 1.64–10.44) compared with the natural cycle. The risks of cesarean delivery (adjusted odds ratio, 1.93; 95% confidence interval, 1.78–2.18), preterm birth (adjusted odds ratio, 1.55; 95% confidence interval, 1.25–1.93), and low birthweight (adjusted odds ratio, 1.42; 95% confidence interval, 1.18–1.73) were also higher for hormone replacement cycles. No significant difference in the risk of congenital anomalies was observed between the 2 cycles.

**CONCLUSION:**

The risk of hypertensive disorders of pregnancy, placenta accreta, cesarean delivery, preterm delivery, and low birthweight was higher in hormone replacement cycles than in natural cycles, whereas the risk of congenital anomalies was similar between both cycles. Further follow-up is needed to investigate these risks and to explore alternative endometrial preparation methods.

**AJOG Global Reports at a Glance**

**Why was this study conducted?**

This study aimed to compare the maternal and obstetrical outcomes between natural and hormone replacement cycles under uniform protocols.

**Key findings**

Hormone replacement cycles were associated with a higher occurrence of hypertensive disorders of pregnancy (adjusted odds ratio, 2.16; 95% confidence interval, 1.66: 95% confidence accreta, cesarean delivery, preterm birth, and low birthweight compared with natural cycles.

**What does this add to what is known?**

The occurrence of pregnancy complications, cesarean delivery, preterm delivery, and low birthweight might be higher in hormone replacement cycles than in natural cycles, whereas the risk of congenital anomalies between both cycles was similar.

## Introduction

The number of frozen embryo transfer (FET) cycles has progressively increased since vitrification techniques were established in 1999 and 2000.^1^^,^^2^ In Japan, 89.4% of births derived from assisted reproductive technologies (ARTs) are obtained from FET cycles.^3^ FET has been enhanced by improvements in the field of cryopreservation and the treatment strategy of elective single-embryo transfers to limit multiple pregnancies.^4^ Using the cryopreservation technique, embryos can be transferred at the optimal time without the detrimental effects that ovarian stimulation exerts on endometrial function; therefore, improved pregnancy outcomes are expected after FETs compared with those after fresh embryo transfers.^5^, ^6^, ^7^

Uterine receptivity and synchronization between the endometrium and embryo are essential for successful embryo implantation^8^; thus, FET should be performed when both the embryo and endometrium are well-prepared. Two types of endometrial preparation protocols are currently used for FETs: hormone replacement (HR) cycle (an artificial cycle) and the natural cycle. The HR cycle is the most commonly used protocol worldwide because it enables flexibility in scheduling the embryo transfers for in vitro fertilization (IVF) centers. However, because endometrial preparation in the HR cycle requires long-term hormonal administration, a greater physical and financial burden is imposed on patients. By contrast, endometrial preparation in the natural cycle needs the least medication; therefore, the physical and financial demand can be reduced despite the inflexibility in scheduling. Recent studies have suggested that endometrial preparation in the natural cycle may improve live birth and decreases early pregnancy loss compared with the HR cycle^9^, ^10^, ^11^; therefore, natural-cycle FETs could be considered an effective strategy to improve live-birth rates. Furthermore, in terms of maternal outcomes, FETs in the HR cycle are reportedly associated with an increased occurrence of hypertensive disorders of pregnancy (HDP), postpartum hemorrhage, placenta accreta, and gestational diabetes mellitus compared with natural cycles.^12^, ^13^, ^14^, ^15^, ^16^ However, these reports lack key information such as the protocol for endometrial preparation in both natural and HR cycles and grade of transferred embryos. Furthermore, the number and stage of transferred embryos were significantly different between the protocols. These factors may result in critical bias when investigating maternal and infant health.

Thus, this study aimed to compare maternal and obstetrical outcomes between the natural and HR cycles under uniform protocols. Furthermore, the outcomes in the natural cycle were stratified by the timing of the transfers and compared to reveal whether the transfer timing affected maternal and obstetrical outcomes. In this study, we retrospectively analyzed maternal and obstetrical outcomes in a single-center 10-year cohort after single vitrified-warmed blastocyst transfers (SVBTs) in natural and HR cycles.

## Materials and Methods

### Study patients

We retrospectively analyzed all clinical records of women who underwent oocyte retrieval during a clomiphene citrate (CC)-based minimal stimulation cycle followed by SVBT in the natural and HR cycles at the Kato Ladies Clinic between January 2008 and December 2017. Only data from patients with singleton pregnancies were included. During the study period, embryo cryopreservation was performed using a vitrification method in all cycles. Complete follow-up data from patients with delivery were used for the analysis of pregnancy complications, and patients with incomplete data were excluded. Follow-up data on obstetrical outcomes were compared among the groups. Data of patients who had cervical incompetence were excluded from the analysis of neonatal outcomes. We classified infertility according to its cause as ovulation (irregular menstruation caused by polycystic ovarian syndrome or diminished ovarian reserve [DOR]), tubal factor (diagnosed by hysterosalpingography), endometriosis (diagnosed by ultrasound), endometrial factor (diagnosed by hysteroscopy), male factor (diagnosed by semen analysis), combined, or unexplained (patients not diagnosed with any cause).

### Ethical approval

This retrospective cohort study was approved by the Institutional Review Board of Kato Ladies Clinic (approval number: 21-14). Written informed consent for the analysis was obtained from all patients.

### Minimal ovarian stimulation cycle in vitro fertilization

A detailed protocol for minimal stimulation with CC has been previously reported.^17^, ^18^, ^19^ Briefly, CC (50–100 mg/d; Fuji Pharma Co, Ltd, Tokyo, Japan) was orally administered from day 3 of the retrieval cycle to the day before induction of final oocyte maturation (days 9–21). Ovulation triggering was performed using a nasal spray containing the gonadotropin-releasing hormone agonist buserelin (Suprecur; Mochida Pharmaceutical Co, Ltd, Tokyo, Japan; or Buserecur; Fuji Pharma Co, Ltd, Tokyo, Japan).

Oocyte retrieval is generally performed 34 to 36 hours after triggering using a 21–22G needle (Kitazato Corporation, Shizuoka, Japan) without anesthesia or follicular flushing. Cumulus–oocyte complexes were collected, washed, and then transferred to a human tubal fluid medium (Kitazato Corporation) with paraffin oil at 5% CO_2_ in air at 37°C for culturing until either conventional IVF was performed 3 hours later,^20^ or in cases of intracytoplasmic sperm injection, denudation was performed 4 hours after oocyte retrieval.^21^^,^^22^ All embryos were cultured at 37°C (gas phase: 5% O_2_, 5% CO_2,_ and 90% N_2_), with 100% humidity in a water jacket or with nonhumidified incubators (Astec Co, Ltd, Fukuoka, Japan). Embryo vitrification and warming were performed using Cryotop (Kitazato Corporation), as described previously.^23^ Briefly, blastocysts were equilibrated in equilibrium solution consisting of 7.5% (v/v) ethylene glycol and 7.5% (v/v) dimethylsulphoxide for 15 minutes. Blastocysts were then transferred to a vitrification solution consisting of 15% (v/v) ethylene glycol, 15% (v/v) dimethylsulphoxide, and 0.5 M sucrose for 1.5 minutes. Next, they were placed on the Cryotop and immediately plunged into liquid nitrogen. For warming, the Cryotop was placed into a warming solution of 1.0 M sucrose at 37°C for 1 minute. The blastocysts were then removed from the warming solution and transferred to a diluent solution of 0.5 M sucrose at room temperature. After 3 minutes, they were transferred to the washing solution without sucrose. For final dilution, the blastocysts were transferred to the washing solution for 1 minute.

### Embryo transfer

The endometrial preparation method was determined after consultation with patients at the initiation of SVBT cycles. In principle, SVBT was performed after confirmation of spontaneous ovulation in the natural cycle. Alternatively, HR cycles were selected in cases of severe ovulatory failure, ovarian insufficiency, luteal insufficiency, or other cases in which SVBT in the ovulatory cycle was deemed inappropriate.

In the natural cycle, ovulation date was estimated from the endometrial thickness, follicle size, and hormonal patterns, or determined by performing buserelin triggering. The timing of SVBT in the natural cycle was classified into 2 patterns according to the timing of ovulation: on day 4.5 (OV 4.5) or day 5 (OV 5.0) after ovulation. When the serum luteinizing hormone (LH) level had not increased on the day of the trigger, SVBT was scheduled on day 7 after the trigger (OV 5.0). When the LH level was slightly increased on the day of trigger, SVBT was scheduled on day 6 after the trigger (OV 5.0). SVBTs were performed as previously described.^24^ Embryo transfer was performed under vaginal ultrasound guidance using a specially designed soft silicone inner catheter (Kitazato ET catheter; Kitazato Corporation) by placing a single embryo in a minimal volume in the upper part of the uterine cavity. Dydrogesterone (Duphaston, 30 mg/d; Mylan EPD G.K., Tokyo, Japan) was administered orally during the early luteal phase after SVBT.

The HR cycle consists of administration of exogenous estradiol and progesterone. Estradiol administration (Estrana, Hisamitsu Pharmaceutical, Co, Inc, Saga, Japan; Julina, Bayer Yakuhin, Ltd, Tokyo, Japan) was initiated on the second day of menstruation. Dydrogesterone administration was initiated after confirmation that the endometrial thickness was >7 mm and serum estradiol level had reached 200 pg/mL. Progesterone was intravaginally administered from 3 days after the initiation of dydrogesterone administration. SVBTs were performed 4 days after the initiation of progesterone administration.

Maternal and neonatal outcomes were obtained from a questionnaire filled out by patients after the infant's 1-month examination. Pregnancy complications were diagnosed in maternity hospitals. All pregnant women were invited to respond to the questionnaire at 9 weeks of gestation, at the second trimester, and after delivery. If they did not respond, we conducted a follow-up regarding their outcomes.

### Study outcomes

The primary outcomes were pregnancy complications and major anomalies. Pregnancy complications include HDP; gestational diabetes mellitus; hemolysis, elevated liver enzymes, and low platelet count (HELLP) syndrome; preterm premature rupture of membranes; low-lying placenta; placenta previa; placenta accreta; and placental abruption. Neonatal outcomes included gestational age (≤27, 28–36, and ≥37 weeks), birthlength, birthweight, small for gestational age (SGA), and large for gestational age (LGA).

The questionnaire requested information on the following: date and mode of delivery, sex, birthweight, birthlength, presence of any birth defect or other anomaly, and pregnancy complications. Live birth was defined as delivery at ≥22 weeks’ gestation. Preterm delivery was defined as delivery occurring at <37 weeks’ gestation. Low birthweight was defined as <2500 g. Perinatal mortality was defined as the sum of stillbirths (≥22 pregnancy weeks) and early (within 7 days) infant deaths. SGA and LGA were defined as birthweights <10th and >90th percentile, respectively, according to the Japanese national reference for neonates.^25^ Congenital anomalies were classified using the International Statistical Classification of Diseases and Related Health Problems, Tenth Revision (ICD-10) codes by reformatting the answers of the parent questionnaires.^26^ Only major congenital anomalies were classified according to the European Surveillance of Congenital Anomalies (EUROCAT) guidelines into the following 13 classes^27^: nervous system, eyes, ears, face, neck, congenital heart defects, respiratory, orofacial clefts, digestive system, abdominal wall defects, urinary, genital, limb, other anomalies or syndromes, and chromosomal. According to the EUROCAT guidelines revised in November 2021, the following cases were not registered: cases of cerebral palsy and cases with only minor defects, excluding cases that were associated with major anomalies.

### Statistical analyses

All statistical analyses were performed using the JMP software (SAS Institute, Cary, NC). Proportion data were analyzed using the chi-square test. Normally distributed continuous parameters were compared using analysis of variance, and statistical significance was determined using Tukey's test for post hoc analysis. When the data were not distributed normally, the Kruskal–Wallis and the Steel–Dwass multiple-comparison test were used. A univariate logistic regression analysis was performed to identify confounders that were potentially associated with maternal and perinatal outcomes (Supplemental Table 1). The likelihood ratio test for the significance of the coefficient was performed, and variables with *P*<.10 were used as confounders. Similarly, a multivariate logistic regression analysis for maternal and perinatal outcomes was used to adjust bias (using covariates and confounders) and verify statistical significance using the Wald chi-square test.^28^ Odds ratios (ORs) and adjusted ORs (AORs) are reported with 95% confidence intervals for each group. The OV 4.5 group was used as the reference for the logistic regression analysis. A *P* value of <.05 was considered significant.

## Results

### Characteristics of the study cohort

In total, 67,018 SVBTs (OV 4.5 group, 29,705 cycles; OV 5.0 group, 31,995 cycles; HR group, 5318 cycles) were performed during the study (Table 1). Maternal age, body mass index, smoking history, number of previous deliveries, and causes of infertility were significantly different among the groups. Culture time and morphology of transferred blastocysts varied among the groups. The serum levels of estradiol and progesterone were significantly different among the groups, although the endometrial thickness was comparable among the groups. Clinical pregnancy rates were significantly different among the groups. The multivariate logistic regression analysis revealed that the AOR for clinical pregnancy in the OV 4.5 group was comparable to that in the OV 5.0 group (Supplemental Table 2). However, the AOR for clinical pregnancy in the HR group was significantly lower than that in the OV 4.5 group.

**Table 1:** Characteristics of the study cohort.

Characteristic	OV 4.5	OV 5.0	HR	*P* value
Embryo transfer cycles, n	29,705	31,995	5318	
Maternal age, mean±SEM	38.2±0.0^a^	38.6±0.0^b^	37.9±0.1^c^	<.0001
Body mass index, mean±SEM	20.7±0.0^a^	20.6±0.0^b^	20.8±0.0^a^	<.0001
Smoking, n (%)	1644 (5.5)^a^	1577 (4.9)^b^	157 (3.0)^c^	<.0001
Previous delivery, n (%)	2549 (8.6)^a^	2427 (7.6)^b^	391 (7.4)^b^	<.0001
Cause of infertility				
Ovulation, n (%)	116 (0.4)^a^	183 (0.6)^b^	151 (2.8)^c^	<.0001
Tubal factor, n (%)	4169 (14.0)^a^	4712 (14.7)^b^	708 (13.3)^a^	.0048
Endometriosis, n (%)	588 (2.0)	629 (2.0)	97 (1.8)	.7498
Endometrial factor, n (%)	270 (0.9)^a^	228 (0.7)^b^	35 (0.7)a,b	.0117
Male factor, n (%)	2985 (10.1)^a^	3387 (10.6)^b^	398 (7.5)^c^	<.0001
Combined, n (%)	3849 (13.0)^a^	3578 (11.2)^b^	789 (14.8)^c^	<.0001
Unexplained, n (%)	17,728 (59.7)	19,278 (60.3)	3140 (59.0)	.1438
Embryo culture time (h), mean±SEM	129.3±0.1^a^	129.8±0.1^b^	130.0±0.2^b^	<.0001
Blastocyst diameter, mean±SEM	183.3±0.1	183.1±0.1	183.0±0.3	.3147
Morphologically good blastocysts, n (%)	10,220 (34.4)^a^	10,745 (33.6)^b^	1573 (29.6)^c^	<.0001
Serum estradiol level (pg/mL), mean±SEM	156.3±0.4^a^	176.0±0.5^b^	201.2±1.3^c^	<.0001
Serum progesterone level (ng/mL), mean±SEM	16.3±0.0^a^	17.1±0.0^b^	10.5±0.1^c^	<.0001
Endometrial thickness (mm)	10.6±0.1	10.4±0.1	10.7±0.3	.3073
Clinical pregnancy, n (%)	15,216 (51.2)^a^	16,016 (50.1)^b^	2500 (47.0)^c^	<.0001
Singleton pregnancy, n	15,095	15,903	2488	—
Follow-up data on the pregnancy	14,950	15,722	2476	—
Miscarriage, n	5324	5752	994	—
Deliveries, n	9626	9970	1482	—

*HR*, hormone replacement; *OV*, ovulation; *SEM*, standard error of the mean.

a–c Different superscript letters indicate a significant difference at *P*<.05.

### Maternal outcomes after single vitrified-warmed blastocyst transfer

The maternal outcomes are described in Table 2. No significant differences in maternal outcomes were observed between the OV 4.5 and OV 5.0 group. Significantly higher rates of HDP and placenta accreta were observed in the HR group than in the OV 4.5 and OV 5.0 groups. The incidence of other complications was comparable among the 3 groups. The multivariate logistic regression analysis also revealed a higher AOR for the incidence of HDP and placenta accreta in the HR group (Table 3).

**Table 2:** Pregnancy complications during the perinatal period

Complication	OV 4.5	OV 5.0	HR	*P* value
Deliveries, n				
Pregnancy complications, n (%)	916 (9.5)^a^	997 (10.0)^a^	190 (12.8)^b^	.0004
Hypertensive disorders of pregnancy, n (%)	287 (3.0)^a^	331 (3.3)^a^	81 (5.5)^b^	<.0001
Gestational diabetes mellitus, n (%)	341 (3.5)	386 (3.9)	62 (4.2)	.3119
HELLP syndrome, n (%)	14 (0.2)	10 (0.1)	3 (0.2)	.4799
Preterm premature rupture of membranes, n (%)	29 (0.3)	27 (0.3)	6 (0.4)	.6633
Low-lying placenta, n (%)	75 (0.8)	63 (0.6)	8 (0.5)	.3526
Placenta previa, n (%)	149 (1.6)	175 (1.8)	24 (1.6)	.5203
Placenta accreta, n (%)	14 (0.2)^a^	12 (0.1)^a^	7 (0.5)^b^	.0056
Placental abruption, n (%)	30 (0.3)	27 (0.3)	4 (0.3)	.8589
Other, n (%)	49 (0.5)	41 (0.4)	11 (0.7)	.1925

*HELLP*, hemolysis, elevated liver enzymes, and low platelet count; *HR*, hormone replacement; *OV*, ovulation.

a,b Different superscript letters indicate a significant difference at *P*<.05.

**Table 3:** Logistic regression analysis of pregnancy complications

Adverse maternal outcomes	Group	OR (95% confidence intervals)	*P* value	aOR^a^(95% confidence intervals)	*P* value
Pregnancy complications^a^	OV 5.0	1.07 (0.97–1.17)	.1743	1.05 (0.95–1.16)	.3133
	HR	1.39 (1.17–1.64)	.0001	1.55 (1.30–1.84)	<.0001
Hypertensive disorders of pregnancy^a^	OV 5.0	1.10 (0.94–1.30)	.2257	1.09 (0.92–1.28)	.2929
	HR	1.84 (1.42–2.38)	<.0001	2.17 (1.67–2.81)	<.0001
Gestational diabetes mellitus^a^	OV 5.0	1.10 (0.95–1.28)	.1786	1.09 (0.94–1.28)	.2210
	HR	1.14 (0.86–0.15)	.3417	1.26 (0.94–1.69)	.1094
HELLP syndrome^a^	OV 5.0	0.64 (0.28–1.43)	.2782	0.66 (0.28–1.51)	.3270
	HR	1.31 (0.37–4.53)	.6684	1.54 (0.42–5.64)	.5125
Preterm premature rupture of membranes^a^	OV 5.0	0.92 (0.53–1.60)	.7855	0.91 (0.52–1.58)	.7428
	HR	1.51 (0.62–3.68)	.7855	1.61 (0.65–3.94)	.2956
Low-lying placenta^a^	OV 5.0	0.86 (0.61–1.21)	.4110	0.83 (0.59–1.17)	.2990
	HR	0.74 (0.35–1.55)	.4372	0.74 (0.35–1.56)	.4360
Placenta previa^a^	OV 5.0	1.15 (0.92–1.43)	.2111	1.12 (0.90–1.41)	.2914
	HR	1.08 (0.70–1.67)	.7096	1.06 (0.67–1.68)	.7809
Placenta accreta^a^	OV 5.0	0.88 (0.40–1.95)	.7700	0.72 (0.33–1.60)	.4336
	HR	3.54 (1.41–8.89)	.0071	3.85 (1.54–9.60)	.0038
Placental abruption^a^	OV 5.0	0.83 (0.48–1.41)	.4965	0.88 (0.52–1.50)	.6585
	HR	0.67 (0.20–2.22)	.5211	0.91 (0.32–2.62)	.8721

Reference: OV 4.5 group.

*aOR*, adjusted odds ratio; *HELLP*, hemolysis, elevated liver enzymes, and low platelet count; *HR*, hormone replacement; *OR*, odds ratio; *OV*, ovulation.

a Confounders: maternal age, body mass index, smoking, previous delivery, cause of infertility, culture time, and blastocyst morphology.

### Obstetrical outcomes after single vitrified-warmed blastocyst transfer

No significant differences in obstetrical outcomes were observed between the OV 4.5 and OV 5.0 group (Table 4). Higher rates of cesarean delivery and preterm delivery were observed in the HR group than in the OV 4.5 and OV 5.0 groups. Shorter birth length and lower birthweight were frequently observed in the HR group compared with the OV 4.5 and OV 5.0 groups. Meanwhile, the incidences of stillbirth, infant death, and birth defects were comparable among the 3 groups. The multivariate logistic regression analysis also demonstrated higher AORs for the incidence of cesarean delivery, preterm delivery, and low birthweight in the HR group (Table 5).

**Table 4:** Obstetrical outcomes stratified by transfer method

Outcomes	OV 4.5	OV 5.0	HR	*P* value
Patients with deliveries, n	9626	9970	1482	
Completed follow-up data on neonatal outcomes, n (%)	9388 (97.5)	9751 (97.8)	1439 (97.1)	.1705
Patients without cervical incompetence, n	9375 (99.9)	9734 (99.8)	1434 (99.7)	.1990
Live birth, n (%)	9340 (99.6)	9701 (99.7)	1431 (99.8)	.6077
Stillbirth, n (%)	35 (0.4)	33 (0.3)	3 (0.2)	.6077
Live birth				
Cesarean delivery rate, n (%)	3087 (33.1)^a^	3219 (33.2)^a^	621 (43.4)^b^	<.0001
Gestational age, wk, mean±SEM	39.1±0.0	39.1±0.0	39.0±0.1	.2103
≤27 wk, n (%)	24 (0.3)	25 (0.3)	8 (0.6)	.1128
28–36 wk, n (%)	481 (5.2)^a^	493 (5.1)^a^	107 (7.5)^b^	.0006
≥37 wk, n (%)	8835 (94.6)^a^	9183 (94.7)^a^	1316 (92.0)^b^	.0001
Birth length, cm, mean±SEM	49.0±0.0^a^	49.1±0.0^a^	48.7±0.1^b^	<.0001
Birthweight, g, mean±SEM	3039.2±4.5	3033.4±4.3	3001±13.1	.0080
Small for gestational age	388 (4.2)	384 (4.0)	70 (4.9)	.2431
Large for gestational age	1569 (16.8)	1528 (15.8)	237 (16.6)	.1410
Infant sex				.0663
Male, n (%)	4805 (51.5)	5055 (52.1)	783 (54.7)	
Female, n (%)	4535 (48.5)	4646 (47.9)	648 (45.3)	
Infant death, n (%)	8 (0.1)	12 (0.1)	3 (0.2)	.3845
Birth defect, n (%)	230 (2.5)	247 (2.6)	38 (2.7)	.8788
Stillbirth				
Birth defect, n (%)	4 (11.4)	3 (9.1)	1 (33.3)	.4452

*HR*, hormone replacement; *OV*, ovulation; *SEM*, standard error of the mean.

a,b Different superscript letters indicate a significant difference at *P*<.05.

**Table 5:** Logistic regression analysis of neonatal outcomes

Adverse neonatal outcomes	Group	OR(95% confidence intervals)	*P* value	aOR(95% confidence intervals)	*P* value
Stillbirth^a^	OV 5.0	0.90 (0.56–1.46)	.6906	0.94 (0.57–1.52)	.8074
	HR	0.55 (0.17–1.82)	.3349	0.64 (0.19–2.10)	.4569
Cesarean delivery^a^	OV 5.0	1.00 (0.94–1.06)	.8480	1.00 (0.94–1.06)	.9516
	HR	1.55 (1.38–1.73)	<.0001	1.94 (1.71–2.19)	<.0001
Preterm delivery (<37 wk)^a^	OV 5.0	0.98 (0.87–1.11)	.8371	0.97 (0.85–1.10)	.6667
	HR	1.52 (1.23–1.88)	<.0001	1.54 (1.24–1.92)	<.0001
Low birthweight (<2500 g)^a^	OV 5.0	1.01 (0.90–1.12)	.8334	1.00 (0.90–1.11)	.9356
	HR	1.36 (1.13–1.64)	.0010	1.43 (1.18–1.73)	.0003
Small for gestational age^a^	OV 5.0	0.95 (0.82–1.09)	.4935	0.95 (0.82–1.10)	.4970
	HR	1.18 (0.91–1.54)	.1984	1.21 (0.92–1.58)	.1591
Large for gestational age^a^	OV 5.0	0.95 (0.85–1.05)	.1502	0.92 (0.85–1.00)	.0532
	HR	0.98 (0.84–1.14)	.8233	0.99 (0.84–1.15)	.9020
Infant death^a^	OV 5.0	1.44 (0.59–3.53)	.4204	1.38 (0.56–3.39)	.4752
	HR	2.45 (0.64–9.24)	.1859	2.53 (0.66–9.65)	.1731
Birth defect^a^	OV 5.0	1.01 (0.86–1.19)	.8797	1.00 (0.83–1.21)	.9431
	HR	1.14 (0.84–1.55)	.3803	1.17 (0.82–1.66)	.3703

Reference: OV 4.5 group.

*aOR*, adjusted odds ratio; *HR*, hormone replacement; *OR*, odds ratio; *OV*, ovulation.

a Confounders: maternal age, body mass index, smoking, previous delivery, cause of infertility, culture time, blastocyst morphology, and infant sex.

### Detailed analysis of congenital anomalies

The incidence of each congenital anomaly was similar among the 3 groups (Table 6 and Supplemental Table 3).

**Table 6:** Congenital anomalies stratified by transfer method

Congenital anomalies	OV 4.5	OV 5.0	HR	*P* value
Live birth, n	9340	9701	1431	
Nervous system, n (%)	17 (0.2)	12 (0.1)	2 (0.1)	.5851
Congenital heart defects, n (%)	114 (1.2)	111 (1.1)	22 (1.5)	.4392
Respiratory, n (%)	2 (0.0)	4 (0.0)	0 (0)	.5800
Orofacial clefts, n (%)	9 (0.1)	12 (0.1)	2 (0.1)	.8104
Digestive systems, n (%)	9 (0.1)	15 (0.2)	1 (0.1)	.4337
Abdominal defects, n (%)	2 (0.0)	1 (0.0)	0 (0)	.7325
Urinary, n (%)	26 (0.3)	29 (0.3)	3 (0.2)	.8356
Genital, n (%)	4 (0.0)	3 (0.0)	2 (0.1)	.0842
Limb, n (%)	16 (0.2)	17 (0.2)	5 (0.4)	.3276
Other congenital abnormalities, n (%)	4 (0.0)	2 (0.0)	0 (0)	.5366
Chromosomal, n (%)	33 (0.4)	49 (0.5)	2 (0.1)	.0850
Stillbirth, n	35	33	3	
Congenital heart defects, n (%)	1 (2.9)	0 (0)	0 (0)	.5936
Orofacial clefts, n (%)	0 (0)	0 (0)	1 (33.3)	.0789
Limb, n (%)	1 (2.9)	0 (0)	0 (0)	.5936
Chromosomal, n (%)	2 (5.7)	3 (9.1)	0 (0)	.7660

*HR*, hormone replacement; *OV*, ovulation.

## Discussion

### Principal findings

This study demonstrated that SVBTs in the HR cycle increase the occurrence of HDP, placenta accreta, cesarean delivery, preterm delivery, and low birthweight compared with that in the natural cycle; however, the rate of congenital anomalies did not change. Furthermore, no difference was observed in perinatal outcomes or congenital anomalies because of a half-day difference in the transfer timing in the natural cycle.

### Results and clinical implications

Given that the risk of SGA did not increase with the increased risk of low birthweight, the latter may have been correlated with the risk of preterm delivery. As for HDP, its risk increased in HR cycles, similarly to what was observed in previous reports.^13^^,^^14^ Some studies have discussed the mechanisms by which the risk of HDP increases during the HR cycle: the corpus luteum (CL), which is not formed in HR cycles, produces not only estradiol and progesterone but also vasoactive products such as relaxin, vascular endothelial growth factor, and angiogenic proxies of estrogen, which regulate the maternal circulatory system in early pregnancy.^12^^,^^29^^,^^30^ Therefore, the lack of CL-derived ligands might be associated with the increased occurrence of HDP in HR cycles. This highlights the importance of the CL in women with regular ovulatory cycles, noting that endometrial preparation through increased estradiol levels from growing follicles, natural LH surges, and CL development in the natural cycle is the preferred approach.^12^

In this study, the risk of placenta accreta was higher in HR cycles than in natural cycles. The reported prevalence of placenta accreta is 0.17%^31^; in this study, it was 3 times higher in HR cycles. Placenta accreta is caused by trophoblast penetration into the myometrium or direct contact between the myometrium and chorionic villi owing to defective formation of the decidua, with a history of uterine surgery being a risk factor.^32^ Unfortunately, history of uterine surgery was not included in the survey and was not analyzed in this study.

### Strengths and limitations

The strength of this study was its analysis of a large dataset from a single center. In addition, the use of drugs for endometrial preparation, techniques of oocyte retrieval and transfer, and culture conditions were uniform. Therefore, potential bias owing to differences in the detailed conditions that potentially occur in multicenter data collection is not likely.

This study also had certain limitations. First, the patient background was not uniform. When transferring a thawed blastocyst, endometrial preparation is performed in the natural cycle, and an HR cycle is selected when endometrial adjustment fails or is expected to fail in the ovulatory cycle because of ovulation failure or DOR. Therefore, differences in patient backgrounds that cannot be adjusted from these data may be biased. One of the several reasons for selecting endometrial adjustment in this study may be DOR considering the patient backgrounds. As to whether DOR worsens perinatal outcomes, a previous study reported no significant difference in perinatal outcomes compared with normal responders,^33^ whereas another study revealed that HDP is more common in DOR; nevertheless, no consensus has been reached.^34^ Further studies are necessary to reveal the influence of ovarian function and reserve on perinatal outcomes. Furthermore, patient preferences and schedules were also considered when the endometrial preparation was decided. Most patients desire to minimalize the use of exogenous hormones to reduce economic and physical burden. Meanwhile, some patients prioritize their schedule and select the HR cycle. These differences in patient preference might have led to bias in this study. Second, this study lacked data on the number of previous ART cycles. Furthermore, data on maternal and obstetrical outcomes were collected using self-reported parental questionnaires. Self-reported maternal and perinatal complications could be potentially erroneous, particularly where uncommon or complex medical terms were involved. Thus, a crosschecking process could have made the data more credible. Moreover, buserelin was used for ovulation trigger in this study; the results might vary when human chorionic gonadotropin is used as the trigger. Therefore, to validate our findings, a comparative study between the HR cycle and natural cycle with human chorionic gonadotropin is required.

### Conclusions

The risk of HDP, placenta accreta, cesarean delivery, preterm delivery, and low birthweight was higher in HR cycles than in natural cycles, whereas the risk of congenital anomalies was similar between the 2 types of cycles. Furthermore, no difference was observed in perinatal outcomes or congenital anomalies because of a half-day difference in transfer timing in the natural cycle. As frozen-thawed transfers become the mainstream method of ART, the HR method of endometrial preparation will become increasingly convenient given that it allows easy schedule adjustment. However, further follow-up is needed to investigate these risks and explore improved endometrial preparation methods.

## Appendix Supplementary materials

Image, application 1

## Footnotes

Supplementary material associated with this article can be found in the online version at doi:10.1016/j.xagr.2022.100081.
