# Supplementary material for: Endometrial preparation and maternal and obstetrical outcomes after frozen blastocyst transfer
Source: AJOG Glob Rep. 2022 Aug 7;2(4):100081. doi: 10.1016/j.xagr.2022.100081 (PMC9664017; doi:10.1016/j.xagr.2022.100081)
Supplement: Supplementary file 1 [file mmc1.docx]

Supplemental Table 1. The *P* value of the univariate logistic regression analysis between the confounders and outcomes.

|  | Maternal age | Body mass index | Smoking | Previous delivery | Cause of infertility | Blastocyst morphology | Infant sex |
| --- | --- | --- | --- | --- | --- | --- | --- |
| Pregnancy complications | *P* < 0.0001 | *P* < 0.0001 | *P* = 0.0147 | *P* = 0.7971 | *P* = 0.0062 | *P* = 0.6852 | – |
| Stillbirth | *P* = 0.2275 | *P* = 0.0129 | *P* = 0.8602 | *P* = 0.0605 | *P* = 0.5818 | *P* = 0.5215 | – |
| Caesarean section | *P* < 0.0001 | *P* < 0.0001 | *P* = 0.1543 | *P* = 0.5750 | *P* < 0.0001 | *P* = 0.0009 | *P* = 0.1753 |
| Preterm delivery | *P* = 0.1027 | *P* < 0.0001 | *P* = 0.3592 | *P* = 0.2838 | *P* = 0.0041 | *P =* 0.4006 | *P* < 0.0001 |
| Low birth weight | *P* = 0.0382 | *P* = 0.0204 | *P* = 0.1561 | *P* = 0.2411 | *P* = 0.1979 | *P* = 0.7569 | *P* < 0.0001 |
| Small for gestational age | *P* = 0.0339 | *P* < 0.0001 | *P* = 0.3037 | *P* = 0.1496 | *P* = 0.7123 | *P* = 0.1188 | *P* = 0.1042 |
| Large for gestational age | *P* = 0.1649 | *P* < 0.0001 | *P* = 0.8697 | *P* = 0.4934 | *P* = 0.0003 | *P* = 0.0022 | *P* = 0.0005 |
| Infant death | *P* = 0.4567 | *P* = 0.6644 | *P* = 0.3944 | *P* = 0.2911 | *P* = 0.8850 | *P* = 0.7794 | *P* = 0.9797 |
| Birth defect | *P* = 0.0451 | *P* = 0.6392 | *P* = 0.0210 | *P* = 0.0051 | *P* = 0.2325 | *P* = 0.5537 | *P* = 0.0519 |

Supplemental Table 2. Logistic regression analysis of clinical pregnancy.

| Group | Odds ratio  (95% confidence intervals) | *P* value |  | Adjusted odds ratio*  (95% confidence intervals) | *P* value |
| --- | --- | --- | --- | --- | --- |
| OV 4.5 | Reference | – |  | Reference | – |
| OV 5.0 | 0.95 (0.97–1.17) | 0.0038 |  | 1.03 (0.99–1.06) | 0.1453 |
| HR | 0.84 (0.79–0.89) | <0.0001 |  | 0.88 (0.82–0.93) | 0.0001 |

Reference: OV 4.5 group. *Confounders: maternal age, body mass index, smoking, previous delivery, cause of infertility, culture time, and blastocyst morphology.

OV, ovulation.

Supplemental Table 3. Details of congenital malformations.

| Anomaly group | EUROCAT subgroups |
| --- | --- |
| Nervous system | Hydrocephalus, spina bifida |
| Eye | Not applicable |
| Ear, face, and neck | Not applicable |
| Congenital heart defects | Double outlet right ventricle, transposition of great vessels, single ventricle, ventricular septal defect, atrial septal defect, tetralogy of Fallot, tricuspid atresia and stenosis, Ebstein’s anomaly, pulmonary valve stenosis, aortic valve atresia/stenosis, mitral valve anomalies, aortic valve atresia/stenosis, coarctation of aorta, total anomalous pulmonary venous return, patent ductus arteriosus as only CHD in term infants (≥37 weeks), |
| Respiratory | Cystic adenomatous malformation of lung |
| Oro-facial clefts | Cleft lip with or without palate, cleft palate |
| Digestive system | Esophageal atresia with or without trachea-esophageal fistula, duodenal atresia, atresia or stenosis of other parts of small intestine, ano-rectal atresia and stenosis, Hirschsprung disease, diaphragmatic hernia |
| Abdominal wall defects | Gastroschisis, omphalocele |
| Urinary | Multicystic renal dysplasia, congenital hydronephrosis, posterior urethral valve and/or prune belly |
| Genital | Hypospadias |
| Limb | Club foot — talipes equinovarus, polydactyly, syndactyly |
| Other anomalies/syndromes | Craniosynostosis, situs inversus, congenital skin disorders, genetic syndromes + microdeletions |
| Chromosomal | Down syndrome, Patau syndrome/trisomy 13, Edward syndrome/trisomy 18, Turner syndrome, Klinefelter syndrome |
